# Fine Tuning ECG Interpretation for Young Athletes: ECG Screening Using Z-score-based Analysis

**DOI:** 10.1186/s40798-024-00775-9

**Published:** 2024-10-23

**Authors:** Jihyun Park, Chieko Kimata, Justin Young, James C. Perry, Andras Bratincsak

**Affiliations:** 1 Department of Pediatrics, University of California San Diego School of Medicine, San Diego, CA USA; 2 Hawaii Pacific Health, Patient Safety & Quality Services, Honolulu, HI USA; 3 Hawaii Pacific Health Medical Group, Hawaii Pacific Health, Honolulu, HI USA; 4 Department of Pediatrics, John A Burns School of Medicine, University of Hawaii, Honolulu, HI USA; 5 Division of Pediatric Cardiology, Stanford University, Stanford, CA USA

**Keywords:** Electrocardiogram (ECG), Athletes, Screening, Left Ventricular Hypertrophy (LVH), Z-score, Sports screening, Hypertrophic cardiomyopathy

## Abstract

**Background:**

Electrocardiograms (ECGs) in athletes commonly reveal findings related to physiologic adaptations to exercise, that may be difficult to discern from true underlying cardiovascular abnormalities. North American and European societies have published consensus statements for normal, borderline, and abnormal ECG findings for athletes, but these criteria are not based on established correlation with disease states. Additionally, data comparing ECG findings in athletes to non-athlete control subjects are lacking. Our objective was to compare the ECGs of collegiate athletes and non-athlete controls using Z-scores for digital ECG variables to better identify significant differences between the groups and to evaluate the ECG variables in athletes falling outside the normal range.

**Methods:**

Values for 102 digital ECG variables on 7206 subjects aged 17–22 years, including 672 athletes, from Hawaii Pacific Health, University of Hawaii, and Rady Children’s Hospital San Diego were obtained through retrospective review. Age and sex-specific Z-scores for ECG variables were derived from normal subjects and used to assess the range of values for specific ECG variables in young athletes. Athletes with abnormal ECGs were referred to cardiology consultation and/or echocardiogram.

**Results:**

Athletes had slower heart rate, longer PR interval, more rightward QRS axis, longer QRS duration but shorter QTc duration, larger amplitude and area of T waves, prevalent R’ waves in V1, and higher values of variables traditionally associated with left ventricular hypertrophy (LVH): amplitudes of S waves (leads V1-V2), Q waves (V6, III) and R waves (II, V5, V6). Z-scores of these ECG variables in 558 (83%) of the athletes fell within − 2.5 and 2.5 range derived from the normal population dataset, and 60 (8.9%) athletes had a Z-score outside the − 3 to 3 range. While 191 (28.4%) athletes met traditional voltage criteria for diagnosis of LVH on ECG, only 53 athletes (7.9%) had Z-scores outside the range of -2.5 to 2.5 for both S amplitude in leads V1-V2 and R amplitude in leads V5-6. Only one athlete was diagnosed with hypertrophic cardiomyopathy with a Z-score of R wave in V6 of 2.34 and T wave in V6 of -5.94.

**Conclusion:**

The use of Z-scores derived from a normal population may provide more precise screening to define cardiac abnormalities in young athletes and reduce unnecessary secondary testing, restrictions and concern.

**Supplementary Information:**

The online version contains supplementary material available at 10.1186/s40798-024-00775-9.

## Key Points

• Athletes had slower heart rate, longer PR interval, greater QRS axis, longer QRS duration, shorter QTc interval, higher peak amplitude of S waves in leads V1 and V2, Q waves in leads III and V6, R waves in leads II, V5, and V6 compared to control subjects.

• However, most of the athletes had ECG variable Z-scores within range of -2.5 and 2.5 (83%) and − 3 and 3 (91.1%), all of which had no identified cardiac pathologies.

• ECG assessment in athletes utilizing Z-scores derived from normal subjects may guide clinical decision making regarding secondary screening.

**Supplementary Information:**

The online version contains supplementary material available at 10.1186/s40798-024-00775-9.

## Background

Preparticipation examination (PPE) with focused history and physical examination is routinely performed for athletes participating in competitive sports. A 12-lead electrocardiogram (ECG) is currently not part of routine PPE, however, the American Heart Association (AHA) recommends obtaining an ECG if there are any signs, symptoms or elements in the history concerning for a possible cardiovascular condition [1]. In fact, the ECG has higher sensitivity and specificity in predicting the presence of a cardiac condition, such as hypertrophic cardiomyopathy, than history and physical examination alone [2, 3].

ECGs in athletes often reveal findings that may differ from ECGs of the general population, such as lower heart rate, prolonged PR interval, and higher QRS and T wave voltages in precordial leads. These ECG findings are related to the physiologic changes that occur due to endurance or high intensity training, and reflect higher vagal tone, increased left or right ventricular dimensions or mass, and an altered repolarization of the ventricles [4, 5]. Typical ECG findings in athletes, consistent with the ‘athlete’s heart’ entity, have been summarized in key publications by European and North American Societies, including the development of the Seattle Criteria, and most recently by Petek et al. [6–9]. These publications also outlined abnormal ECG findings in athletes that may warrant further investigation for underlying pathologies, such as right bundle branch block, prolonged QTc interval, and right or left axis deviation. Molinari et al. examined ECGs of children aged 3–14 years participating in noncompetitive sports, published means of a few ECG variables in these young athletes, and identified some abnormal ECG findings in a small number of study participants [10]. However, to our knowledge, there has been no methodical comparison of multiple ECG variables in collegiate athletes to non-athletes. In addition, none of the prior publications utilized Z-scores derived from normal population to assess the degree of abnormalities noted on the ECGs in athletes. The objectives of our study were to compare over one hundred ECG variables in collegiate athletes to Z-scored normative standards derived from non-athlete control subjects, delineate the significant differences between the groups, and to define the portion of athletes with ECG variables falling outside the normal range of age and sex-matched control subjects.

## Methods

### Study Design

ECGs and medical records were reviewed retrospectively for collegiate athletes and age and gender matched pediatric and young adult patients aged 17–22 years between January 1st, 2014 to December 31st, 2020. The study received Institutional Review Board approval.

### Study Population

The athlete group consisted of collegiate athletes participating in competitive sports at the University of Hawai‘i between 2014 and 2020. ECGs were obtained as part of routine PPE mandated by the University. The PPE included screening questionnaires with specific questions regarding medical, personal, and family history of athletes, incorporating the 14-element AHA recommendations for preparticipation cardiovascular screening of competitive athletes (Supplementary Table 1) [11]. Supplementary Table 1 outlines specific questions asked on the screening questionnaire and number of athletes with relevant positive answers for each question. Student athletes with abnormal ECG and/or screening questionnaire were referred to cardiology for further assessment.

**Table 1:** Demographics of study subjects by age and sex

		Athlete			Control		
		Female	Male	Total	Female	Male	Total
Age	17 years old	31	13	44	750	466	1216
	18 years old	161	128	289	704	385	1089
	19 years old	54	61	115	646	347	993
	20 years old	44	69	113	689	327	1016
	21 years old	23	48	71	690	375	1065
	22 years old	11	29	40	756	399	1155
Total		324	348	672	4235	2299	6534

The control group consisted of patients with an ECG done at Hawaii Pacific Health medical facilities and Rady Children’s Hospital San Diego between 2007 and 2020, in various settings, including inpatient, outpatient, and emergency department. This validated cohort consisting of subjects with no known heart condition has been previously published and used to define normal values for respective age groups [12]. However, additional patients were included from years 2016 to 2020. In summary, ECGs were obtained for evaluation for heart murmur, irregular heartbeat, syncope, dizziness, brady or tachycardia, fever, and preparticipation screening for non-collegiate sports and for certain diseases [12]. Exclusion criteria for control group included following: (a) missing demographic information; (b) known cardiovascular conditions including channelopathies, or history of cardiac surgery; (c) pregnant women; (d) erroneous ECG value or value that was clearly an outlier, such as R axis > 200 or <-30 degrees, PR > 250 msec, QRS > 150 msec, QTc > 550 msec, R in V1 > 5 mV, R in V6 > 5mV. First record was used in the analysis if multiple ECGs were available for a single patient.

### ECG Measurements

12-lead ECGs (leads I, II, III, aVR, aVL, aVF, V1, V2, V3, V4, V5, V6) were obtained in resting supine position using GE MAC 5500 HD ECG systems (General Electrics, Houston, TX) at 500 Hz sampling frequency. Standardized voltage (10 mm = 1 mV) and speed (25 mm/s) were used. ECGs were excluded from the analysis if they had technical issues, including lead reversal, having a poor baseline, or missing lead information.

### Variables and Analysis

Standard demographic variables such as age and sex were obtained. Values for the ECG variables were generated electronically and exported from the GE Muse v9 system (General Electrics, Houston, TX). The following ECG variables were analyzed in all study subjects: RR interval; PR interval; QRS duration; QT interval, corrected QT interval (QTc) calculated from automated Bazett method; peak amplitudes of P and T waves in leads I, II, III, aVF, V1, V6; peak amplitudes of Q, R, and S waves in all leads; P, R, and T axes; QRS integral; and T wave integral. Subjects were divided into different groups by age and sex as described in our previous report [12].

### Statistical Analysis

Processing and analysis of ECG data, as well as development of normative standards for each age group were described before [12]. In short, numerical data of ECG variables were extracted from the GE Muse v9 ECG acquisition system. Distribution and characteristics of ECG variables were calculated for each age group and sex. Data from athletes and control subjects was compared using Mann-Whitney test, since most of the data did not show typical normal distribution and because there was significant difference between the number of study and control subjects. SAS software version 9.4 (SAS institute, Inc, Cary, NC) and SPSS software version 28.0.1.1 (14) (IBM Corporation, Armonk, NY) were used for statistical analysis.

## Results

The analyzed cohort consisted of 7206 subjects, 672 athletes and 6534 control subjects (Table 1).

### Comparison of Selected ECG Variables in Athletes and Control Subjects

Over one hundred ECG variables were evaluated [12], and of those, 17 ECG variables with clinical significance were selected for further analysis.

**Fig. 1:**
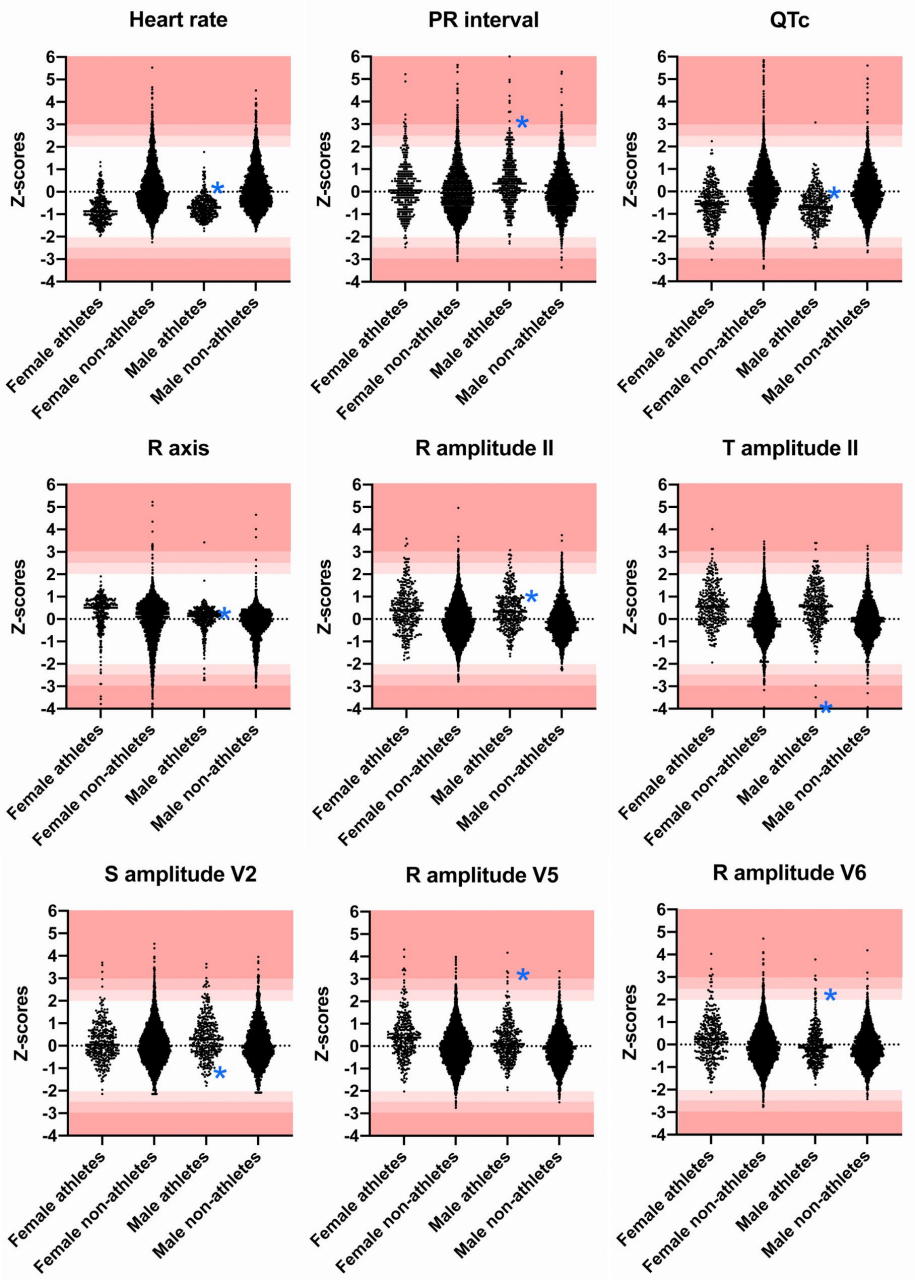
**Distribution of Z-scores of clinically significant ECG variables in control and athletes.** Z-scores of heart rate were lower in athletes than in non-athletes while Z-scores of PR interval were higher in athletes than in non-athletes. Z-scores of R axis were higher in athletes than in non-athletes, representing more rightward axis in athletes. Z-scores of R amplitude in leads II and V5, S amplitude in lead V2 were higher in athletes than in non-athletes. Blue asterisks mark Z-scores for each variable in the athlete who was diagnosed with hypertrophic cardiomyopathy

Athletes had slower heart rate, longer PR interval, greater QRS axis (more rightward), longer QRS duration, and shorter QTc interval compared to control subjects (Table 2; Fig. 1). There was no significant difference between various ages among the athletes, except for the PR interval, with older age being associated with slightly increased PR interval (median 142 msec, IQR 132–157 msec in 17yo, median 150 msec, IQR 138–164 msec in 18-21yo, vs. median 155 msec, IQR 142–179 msec in 22yo, *p* = 0.056). Supplementary Fig. 1 demonstrates distribution of values for ECG variables in controls and athletes by age.

Athletes had higher peak amplitude of S waves in leads V1 and V2, Q waves in leads III and V6, R waves in leads II, V5, and V6 compared to control subjects (Table 2; Fig. 1). Athletes also had higher peak amplitude and larger area of T waves compared to the control subjects in leads II and aVF. Male athletes had higher peak amplitude of T waves in lead V6 compared to females (median 437 uV, IQR 322–567 uV for males; median 346 uV, IQR 273–449 uV in females, p = < 0.001), but there was no significant difference among different age groups (median 400 uV, IQR 296–477 uV in 17yo, median 380 uV, IQR 288–512 uV in 18-21yo, median 383 uV, IQR 301–464 uV in 22yo, *p* = 0.332). Distribution of Z-scores of clinically significant ECG variables in controls and athletes by age and sex are demonstrated in Supplementary Fig. 2.

**Table 2:** Comparison of clinically relevant ECG^1^ variables in athletes and controls

ECG Variable	Lead	Athlete (*N* = 672)	Control (*N* = 6534)	*P* value
		Median (IQR^2^)	Median (IQR)	
HR (beats per minute)	II	61 (54–69)	77 (66–91)	< 0.0001
PR interval (msec)	II	150 (138–164)	144 (132–156)	< 0.0001
R axis (degrees)	II	78 (65–86)	69 (51–81)	< 0.0001
QRS duration (msec)	II	92 (86–98)	86 (80–94)	< 0.0001
QTc (msec)	II	409 (384–426)	428 (411–445)	< 0.0001
T peak amplitude (mV)	II	0.42 (0.31–0.54)	0.3 (0.21–0.40)	< 0.0001
	aVF	0.27(0.18–0.38)	0.17 (0.10–0.25)	< 0.0001
	V6	0.38 (0.29–0.50)	0.30 (0.21–0.42)	< 0.001
T area	II	2332 (1752–2941)	1853 (1311–2554)	< 0.0001
	aVF	1517 (963–2066)	1064 (613–1623)	< 0.0001
S peak amplitude (mV)	V1	0.90 (0.63–1.17)	0.84 (0.59–1.11)	< 0.0001
	V2	1.35 (0.97–1.80)	1.14 (0.82–1.53)	< 0.0001
Q peak amplitude (mV)	III	0.07 (0-0.16)	0.05 (0-0.12)	0.0013
	V6	0.06 (0.03–0.11)	0.04 (0-0.08)	< 0.0001
R peak amplitude (mV)	II	1.46 (1.16–1.79)	1.19 (0.93–1.48)	< 0.0001
	V5	1.64 (1.31–2.03)	1.35 (1.06–1.71)	< 0.0001
	V6	1.36 (1.10–1.64)	1.22 (0.97–1.49)	< 0.0001
S peak amplitude + R peak amplitude (mV)	SV1+ RV6	2.29 (1.83–2.76)	2.07 (1.64–2.53)	< 0.0001
**R’ Measurements**		**Athlete (** ***N*** ** = 157; 23.4%)**	**Control (** ***N*** ** = 1094; 16.7%)**	
R’ peak amplitude (mV)	V1	0.16 (0.10–0.30)	0.13(0.07–0.21)	< 0.0001
R’ area	V1	106 (49–268)	101 (46–220)	< 0.0001
R’ duration (msec)	V1	26 (15–35)	25 (18–34)	< 0.0001

^1^ ECG: electrocardiogram

^2^ IQR: interquartaile range

Among those athletes and control subjects, who had an R’ in V1 (suggestive of incomplete or complete right bundle branch block), the R` peak amplitude, area and duration were larger in athletes compared to the control group. There was no significant difference among different ages for R’ peak amplitude and duration among the athletes.

### ECG Variables in Athletes Being Outside the Normal Range Defined by Z-Scores Derived from Age and Sex-Matched Control Subjects

Using the Z-scored normative standards derived from control subjects [12], most of the ECG variables in athletes fell within normal limits, including heart rate, QRS duration, QRS axis and QTc (Table 3; Fig. 1). There were 117 athletes (17.4%) with ECG Z-score values outside the normal range of -2.5 to 2.5. Z-scores exceeding 2.5 were found in 29 (4.32%) athletes for PR interval, 14 (2.08%) and 12 (1.79%) athletes for T wave peak amplitude in leads II and aVF respectively, and 17 (2.53%), 17 (2.53%) and 13 (1.93%) athletes for R wave peak amplitude in leads II, V5, and V6 respectively (Table 4). Only 60 athletes (8.9%) had Z-score values beyond − 3 and 3 range, and only 5 athletes (0.7%) had Z-score values outside the − 5 and 5 range. Z-scores beyond − 5 to 5 range were found in T amplitude in lead V6 (*n* = 1, Z-score <-5), and PR interval (*n* = 4, Z-score > 5). There was no significant correlation between the type of sports, duration of sports participation (years) and duration of training (hours/week) to ECG variables with Z-scores outside the normal range.

**Table 3:** Athletes and non-athletes with Z-scores outside the range of -2.5 and 2.5, > 3 and > 5

ECG Variable (Lead)	Z score	Athletes (%)	Control (%)	*P*-value
Heart rate (II)	> 2.5	1 (0.15)	173 (2.65)	< 0.001
	> 3	0 (0)	76 (1.16)	
	> 5	0 (0)	2 (0.03)	
PR Interval (II)	> 2.5	29 (4.32)	138 (2.11)	< 0.001
	> 3	16 (2.38)	73 (1.12)	
	> 5	4 (0.60)	17 (0.26)	
R axis	<-2.5	8 (1.19)	81 (1.24)	0.708
	> 2.5	1 (0.15)	22 (0.34)	
	<-3	3 (0.45)	31 (0.47)	
	> 3	1 (0.15)	16 (0.24)	
	<-5	0 (0)	0 (0)	
	> 5	0 (0)	2 (0.03)	
QRS Duration (II)	> 2.5	6 (0.89)	26 (0.40)	0.136
	> 3	2 (0.30)	14 (0.21)	
	> 5	0 (0)	2 (0.03)	
QTc (II)	<-2.5	3 (0.45)	21 (0.32)	0.011
	> 2.5	1 (0.15)	104 (1.59)	
	<-3	1 (0.15)	7 (0.11)	
	> 3	1 (0.15)	63 (0.96)	
	<-5	0 (0)	1 (0.02)	
	> 5	0 (0)	25 (0.38)	
T Peak Amplitude (II)	<-2.5	2 (0.30)	25 (0.38)	< 0.001
	> 2.5	14 (2.08)	26 (0.40)	
	<-3	1 (0.15)	5 (0.08)	
	> 3	6 (0.89)	8 (0.12)	
	<-5	0 (0)	0 (0)	
	> 5	0 (0)	0 (0)	
T Peak Amplitude (aVF)	<-2.5	2 (0.30)	15 (0.23)	< 0.001
	> 2.5	12 (1.79)	19 (0.29)	
	<-3	0 (0)	5 (0.08)	
	> 3	3 (0.45)	5 (0.08)	
	<-5	0 (0)	0 (0)	
	> 5	0 (0)	0 (0)	
T Peak Amplitude (V6)	<-2.5	1 (0.15)	17 (0.26)	< 0.001
	> 2.5	25 (3.72)	85 (1.30)	
	<-3	1 (0.15)	15 (0.23)	
	> 3	15 (2.23)	45 (0.69)	
	<-5	1 (0.15)	4 (0.06)	
	> 5	0 (0)	3 (0.05)	
S Peak Amplitude (V1)	> 2.5	7 (1.04)	88 (1.35)	0.509
	> 3	1 (0.15)	38 (0.58)	
	> 5	0 (0)	0 (0)	
S Peak Amplitude (V2)	> 2.5	15 (2.23)	69 (1.06)	0.007
	> 3	6 (0.89)	33 (0.51)	
	> 5	0 (0)	1 (0.02)	
R Peak Amplitude (II)	> 2.5	17 (2.53)	39 (0.60)	< 0.001
	> 3	4 (0.60)	10 (0.15)	
	> 5	0 (0)	0 (0)	
R Peak Amplitude (V5)	> 2.5	17 (2.53)	44 (0.67)	< 0.001
	> 3	10 (1.49)	18 (0.28)	
	> 5	0 (0)	1 (0.02)	
R Peak Amplitude (V6)	> 2.5	13 (1.93)	38 (0.58)	< 0.001
	> 3	7 (1.04)	15 (0.23)	
	> 5	0 (0)	2 (0.03)	

**Table 4:** Athletes referred to cardiology for abnormal ECG^1^

Abnormal ECG finding	*N*	ECG Variable (Lead)	Z-score median (IQR)	Abnormal echo
Right bundle branch block	9	R’ peak amplitude (V1)		3*^†§^
		R’ area (V1)		
		R’ duration (V1)		
Left axis deviation	9	R axis	-2.72 (-3.47 - -2.62)	2*^†§^
Right axis deviation	4	R axis	2.27 (1.48–2.96)	0
Left ventricular hypertrophy	20	S peak amplitude (V1)	0.46 (0.06–1.42)	3*
		S peak amplitude (V2)	1.43 (0.55–2.16)	
		R peak amplitude (II)	1.67 (0.96–2.13)	
		R peak amplitude (V5)	1.80 (1.20–2.39)	
		R peak amplitude (V6)	1.83 (1.36–2.14)	
Prolonged QTc	2	QTc (II)	1.54 (1.40–1.67)	1^†^
Low atrial rhythm	5	P axis	-4.12 (-4.35-0.60)	3*^†§^
Negative T waves in inferior/lateral leads	9	T peak amplitude (II)	-1.46 (-2.23 - -1.80)	2*
		T peak amplitude (aVF)	-2.01 (-2.23 - -1.80)	
		T peak amplitude (V1)	0.37 (-0.03-1.04)	
		T peak amplitude (V6)	-1.55 (-1.96 - -1.24)	
1st degree AV block^1^	2	PR interval (II)	6.52 (5.28–7.76)	1*
Prolonged QRS	1	QRS duration	1.92	1*
PVC^2^	2			0

*Left ventricular hypertrophy, †Left ventricular dilation, §Right ventricular dilation

^1^ECG: Electrocardiogram

^2^AV block: Atrioventricular block

^3^PVC: Premature ventricular contraction

### Athletes Referred to Cardiology

**Fig. 2:**
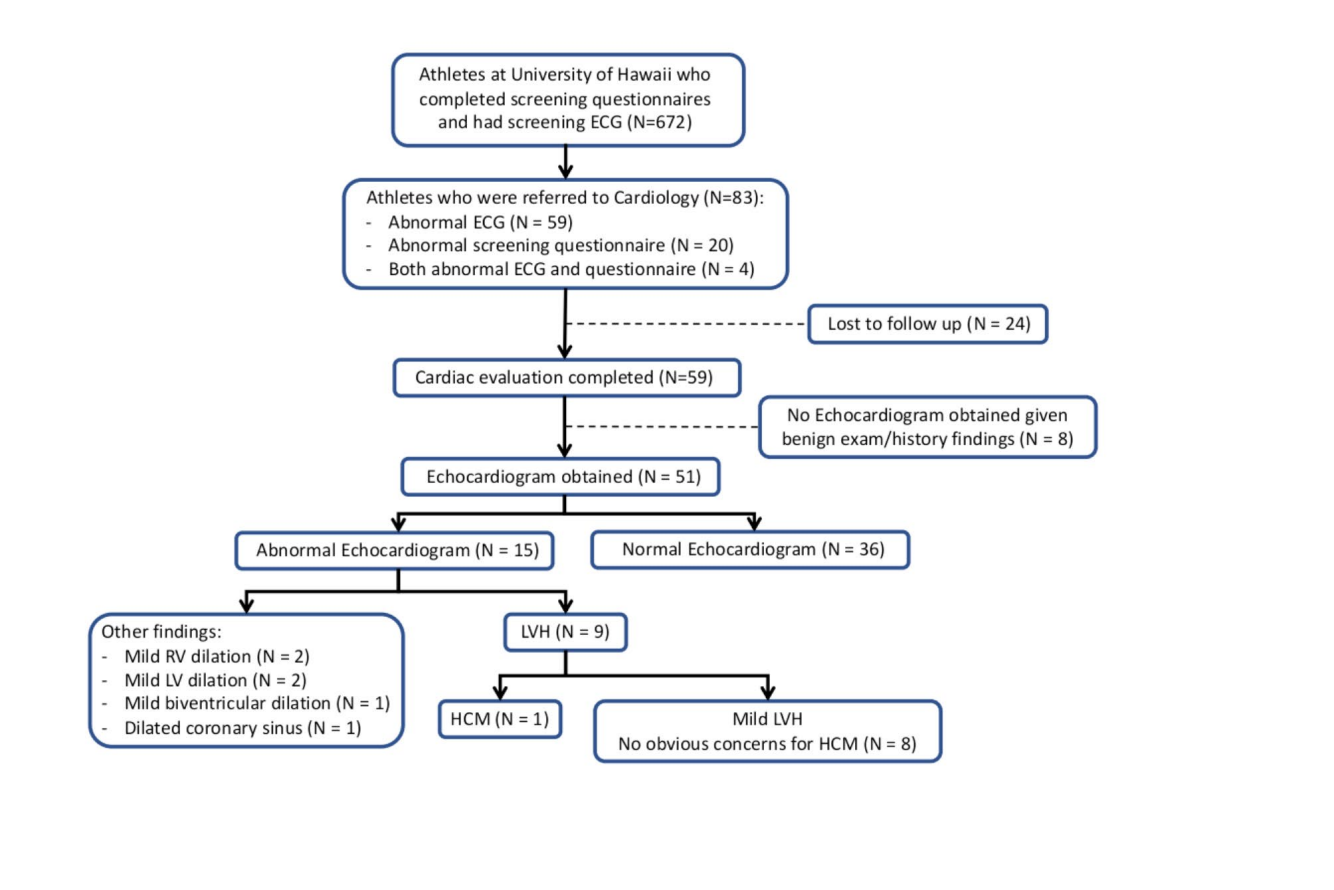
**Athletes referred to cardiology.** Eighty-three athletes were referred to Cardiology due to abnormal questionnaire or ECG findings. Of those, fifty-nine athletes had cardiology visit and fifty-one athletes had echocardiogram completed. Only nine athletes had mild left ventricular hypertrophy on echocardiogram, including one patient with genetically proven diagnosis of hypertrophic cardiomyopathy. (ECG electrocardiogram, LVH left ventricular hypertrophy, RV right ventricle, LV left ventricle, HCM hypertrophic cardiomyopathy)

Eighty-three athletes (12.4% of total of athletes, 24.1% female) were referred to cardiology, 20 for abnormal screening questionnaire, 59 for abnormal ECG findings, and 4 for both abnormal screening questionnaire and abnormal ECG findings (Fig. 2). ECG abnormalities for referral are listed in Table 4. From the 83 referred athletes, 59 completed cardiology evaluation. Referred athletes participated in various sports for a median of 10 years (5–16 years) (Supplementary Table 2). The median duration of weekly training was 12.3 h (ranging 2–25 h/week) (Supplementary Table 2). Despite the wide range in duration of weekly training, majority of athletes (*N* = 574, 85.4%) participated in training at least 10 h per week with small number of athletes (*N* = 17, 2.5%) participating in training for less than 5 h per week. There was no significant correlation between the type of sports, duration of sports participation (years) and duration of training (hours/week) with being referred to cardiology or with a particular ECG abnormality.

A total of 20 (2.98%) athletes were referred for concerns of left ventricular hypertrophy. Z-scores exceeded 2.5 for R waves in leads V5, V6, and II in 5, 4, and 3 athletes, respectively. And 0 and 3 athletes and Z-scores exceeding 2.5 for amplitude of S waves in leads V1 and V2, respectively (Table 5). Of those, 4 athletes had normal echocardiograms despite having Z-scores exceeding 2.5 for R or S wave amplitude in two different leads, while 3 had mild LVH on echocardiogram, but with normal diastolic indices (Table 5). A total of 9 (0.15%) athletes were referred for negative T waves in inferior/lateral leads and only one had mild LVH on echocardiogram (Table 5). The single athlete with a T wave in V6 Z-score < -5 was diagnosed with a cardiomyopathy.

**Table 5:** Z-scores for ECG^1^ variables and corresponding echocardiogram findings for subjects referred due to LVH and negative T waves in inferior/lateral leads

Abnormal ECG findings	*N*	Echocardiogram findings	Z-score				
			S (V1)	S (V2)	R (II)	R (V5)	R (V6)
Left ventricular hypertrophy	20	Mild LVH^2^, HCM^3^	-0.87	-0.97	1.17	3.34	2.36
		Normal	-0.60	3.01	3.08	2.25	1.77
		Normal	2.39	2.18	1.30	1.38	1.12
		Normal	2.01	2.03	1.02	1.37	1.53
		Normal	2.36	0.83	2.07	3.08	2.87
		Mild LVH	0.37	1.39	1.94	1.93	2.91
		Normal	0.50	-0.02	2.29	1.75	2.04
		Normal	0.36	1.46	1.82	0.50	1.88
		Normal	-0.29	0.64	1.70	4.17	3.07
		N/A*	1.33	2.22	0.93	3.27	2.06
		Normal	-0.33	-0.32	0.38	1.95	1.62
		Mild LVH	2.10	3.48	2.41	0.69	0.16
		N/A*	0.17	0.98	0.32	1.84	1.06
		N/A*	0.92	1.64	1.63	2.17	1.44
		Normal	-0.38	1.51	1.94	0.55	2.06
		Normal	1.70	0.28	2.53	0.57	2.01
		Normal	1.07	0.11	2.66	1.65	3.78
		N/A*	0.59	2.63	0.83	1.71	0.86
		Normal	0.42	0.71	-1.33	-0.60	0.11
		Normal	0.39	2.15	0.97	2.80	1.66
			**T (II)**	**T(aVF)**	**T (V1)**	**T (V6)**	
Negative T waves in inferior/lateral leads	9	Normal	-1.31	-1.59	0.37	-1.55	
		Normal	-1.03	-2.01	2.46	-0.91	
		Mild LVH, HCM	-3.49	-2.70	0.79	-5.94	
		Normal	-1.18	-1.55	-0.56	-1.24	
		Normal	-2.97	-2.51	1.04	-1.96	
		Normal	-1.94	-1.96	-0.03	-1.42	
		Normal	-1.46	-1.80	1.37	-1.55	
		Normal	-0.63	-2.23	-0.20	-1.15	
		Normal	-1.91	-2.15	0.36	-2.15	

*Some athletes did not have echocardiograms performed. A few athletes did not follow up with cardiology despite referral and a few athletes did not have echocardiograms per physician discretion

^1^ ECG: Electrocardiogram

^2^ LVH: Left ventricular hypertrophy

^3^HCM: Hypertrophic cardiomyopathy

In the entire cohort there was only one athlete with a cardiac pathology: hypertrophic cardiomyopathy. His work-up was triggered by the abnormal ECG. His ECG showed abnormal negative T waves with a peak amplitude of -0.52 mV in lead II (Z-score − 3.49), -0.41 mV in lead aVF (Z-score − 2.70), -1.13 mV in lead V6 (Z-score − 5.94), mildly prominent R waves with a peak amplitude of 4.27 mV in lead V5 (Z-score 3.34), and 2.90 mV in lead V6 (Z-score 2.36), and normal S wave amplitudes of 0.30 mV in lead V1 (Z-score − 0.87) and 0.51 mV in lead V2 (Z-score − 0.97). The echocardiogram and cardiac MRI showed mild thickening of the left ventricular wall. Genetic testing revealed a pathologic variant in the MYL3 gene. No other athletes that were referred and completed cardiac evaluation (*n* = 59) had identifiable cardiac conditions at the time of follow up (average follow-up time 6.5 years).

### Athletes Meeting Voltage Criteria by Sokolow-Lyon Index for LVH

Total of 191 athletes (28.4%) met voltage criteria for LVH by having sum of peak amplitudes of S wave in lead V1 and R wave in leads V5 or V6 to be greater than or equal to 35 mm based on Sokolow-Lyon Index (Table 6). Of those, only 53 athletes (7.89%) had Z scores > 2.5 in at least one of the following ECG variables: S wave amplitude (leads V1, V2) and R wave amplitude (leads II, V5, V6), and only 21 athletes (3.1%) had Z-score values beyond 3. Of those that had echocardiogram obtained for signs of LVH (*n* = 27), only 6 athletes had LVH by echocardiogram. Three athletes had mild LVH by echocardiogram, who were predicted to have LVH by Sokolow-Lyon index, but no LVH based on Z-score being within − 2.5 to 2.5 range. Both the Sokolow-Lyon index and the Z-scored based screening predicted left ventricular hypertrophy in the patient with hypertrophic cardiomyopathy.

**Table 6:** Number of patients with LVH^1^ diagnosis based on Sokolow-Lyon Index voltage criteria and Z-scores $\:\ge\:$2.5 for S amplitude (leads V1, V2), R amplitude (leads II, V5 and V6)

		S (V1) + *R* (V5) or *R* (V6) $\:\ge\:$ 35 mm	Z score $\:\ge\:$2.5	Both	LVH by echocardiogram
Age (years)	17	1	1	1	0
	18–21	90	48	26	4
	22	10	4	4	2
Total		191(28.42%)	53 (7.89%)	31 (4.61%)	6 (0.89%)

^1^ LVH: Left ventricular hypertrophy

## Discussion

Comparison of over one hundred ECG variables in athletes to age and sex-matched control subjects revealed that despite significant differences between the values of ECG variables in athletes and non-athletes, 83% of athletes had all ECG variables within the normal range, defined by Z-score ≥-2.5 and ≤ 2.5 based on a large cohort of normal subjects. After excluding athletes with mildly increased Z-scores (Z-score -3 to -2.5 and 2.5 to 3) in ECG variables typically associated with “athlete’s heart” (such as HR, PR interval, R peak amplitude in lead II, V5 or V6, S wave peak amplitude in lead V1 or V2), only 71 athletes (10.6%) had “abnormal ECGs” defined by a Z-score outside the normal range. Typical ECG markers of left ventricular hypertrophy were inconsistent in predicting LVH. The only individual with hypertrophic cardiomyopathy had T wave and R wave amplitudes in several leads outside the normal range of Z-scores of -2.5 to 2.5. All athletes with normal Z-scores had no evidence of cardiac arrest or pathology with an average follow-up time of 6.5 years.

### Normal Range of Z-Scores

Z-scores between − 2 and 2 includes 95% of the population, and are generally considered as normal [13]. On the other hand, Z-scores outside of -3 and 3 range are considered abnormal or “highly unusual” as 99.7% of population is included in the − 3 to 3 range [14]. Given that the goal of this study was to identify significant difference between ECG findings in non-athletes and athletes, we defined normal Z-score range to be -2.5 to 2.5 which included 98.7% of population, and ensuring the highly unusual values to be appropriately identified as abnormal [14].

### Athlete ECGs Assessed by Normal Values Based on Non-Athlete Subjects

Differentiating pathologic abnormalities and physiologic changes on athletes’ ECGs helps us determine the necessity of cardiology referral and further diagnostic work-up [6–9]. Our study utilized standardized Z-scores derived from age and sex-matched non-athletic population of young adults to assess how ECG variables of collegiate athletes would fall on a standardized curve. In general, ECG variables in athletes were significantly different from normal non-athlete subjects, consistent with numerous prior publications defining the entity “athlete’s heart”. Athletes’ ECGs showed lower heart rate, longer PR interval, rightward QRS axis, longer QRS duration, shorter QTc duration, taller S and R waves and even taller T waves and larger T wave areas compared to normal subjects. Nevertheless, most athletes (*n* = 555, 82.6%) had ECGs with variables that fell inside the normal range defined by the normal subjects. Among these athletes with normal ECG variables, there was no individual diagnosed with a cardiac abnormality or sudden cardiac arrest/death during an average follow-up of 6.5 years. This observation allows us to postulate that ECG variables derived from normal subjects could be used for athletes to screen for cardiac abnormalities without requiring a large portion of athletes to undergo unnecessary secondary, more intense and costly diagnostic work-ups.

### ECG Markers of Left Ventricular Hypertrophy

Among the athletes referred for further work-up, most of them were evaluated for left ventricular hypertrophy suggested by ECG readings. Among athletes referred for LVH on ECG, 18 met the Sokolow-Lyon criteria for LVH and 11 had a Z-score exceeding 2.5 for one or more ECG variables for LVH (R peak amplitude in leads II, V5, V6 and S peak amplitude in leads V1 and V2) [15]. However, of those that had ECG findings meeting Sokolow-Lyon criteria or high Z-scores for specific variables, only 3 athletes had LVH on echocardiograms, the current gold standard modality defining LVH. There was no single ECG variable predicting left ventricular hypertrophy or dilation, consistent with previous findings among non-athlete subjects [16]. On the contrary, within this cohort of athletes referred to cardiology evaluation, there was no athlete with a diagnosis of LVH by echocardiogram, who did not have abnormal Z-scores on ECG. In addition, the Z-score based screening predicted left ventricular hypertrophy in the patient with hypertrophic cardiomyopathy (HCM).

While neither voltage criteria for LVH nor Z-score for a single ECG variable alone can accurately predict echocardiographic diagnosis of LVH, when combined, it may be useful in determining which athletes may need further cardiology evaluation for abnormal ECG concerning for LVH. If at least one of the variables is exceeding Z-score of 2.5 or less than Z-score of -2.5, it may be beneficial to obtain an echocardiogram for screening, as they are more likely to have abnormality on echocardiogram. If there are 3 or more variables with Z-score values outside of -2.5 to 2.5 range, or if there are Z-score values outside of -3 to 3 range even in a single variable, cardiology evaluation should be strongly considered. The single individual, who had a Z-score outside − 5 to 5 range in an ECG variable other than PR interval, was diagnosed with HCM.

While ECG findings suggesting “athlete’s heart” are already widely known, there are often equivocal findings, such as “borderline ECG findings” as defined by 2017 consensus statement possibly concerning for cardiac pathologies [6]. In addition, different levels of expertise and various comfort level among physicians may decrease the accuracy and increase the inter-rater variability of ECG interpretation [17]. Because of the uncertainties caused by the interpretation of borderline ECG findings and the lack of specialists available for expert interpretation, comprehensive and well-organized ECG screening for collegiate athletes is not mandated and is only done at a few centers of the United States. Our study demonstrated that utilization of Z-scored standards derived from normal subjects may be useful in automated ECG screening of athletes. For an example, a few of the “borderline ECG findings” as defined by 2017 consensus statemen, such as left axis deviation with R axis less than − 30 degrees has Z-score >-2.5 for female and Z-score >-2.0 for male collegiate people aged > 21 yo, while right axis deviation with R axis greater than 120 degrees may still be within Z-score 1–2 range for both females and males, which can be helpful in determining whether such finding in isolation could have more clinical significance or not [6]. Z-score values within normal range can immediately reassure the provider and would limit unnecessary referrals. The degree and number of abnormal variables may guide physicians to pursue further work-up in athletes presenting with abnormal ECG findings. Using Z-score based standards could also limit unnecessary referrals with a higher specificity than historical markers, such as the Sokolow-Lyon index.

### Limitations

Our study was limited by being a retrospective analysis, and not having every athlete undergo secondary screening with echocardiograms. There was also a class imbalance between the athletes and non-athletes affecting the statistical analysis. Additionally, although studied athletes were excluded from the control group, subjects in the control group were not excluded based on the level of physical activity. Furthermore, control group had ECGs performed not only during outpatient setting, but also during acute care setting in the emergency department and inpatient. Their acutely ill state may have affected ECG findings in this control group. In addition, there was only 6.5 years of follow-up data available to identify if subjects that initially presented with abnormal ECG findings had later developed more significant abnormalities on ECGs or other cardiac symptoms or signs concerning for pathologic cardiac conditions. And for the athletes who had benign cardiac evaluation during their appointment, no further follow ups were arranged. Therefore, it is possible that these athletes may have had later onset arrhythmias or other cardiac events requiring care outside of Hawaii Pacific Health, that may have not been identified during initial cardiology visit. Furthermore, African-American athletes are reported more likely to have certain ECG abnormalities compared to athletes with different race. However, our study was limited in that it did not investigate different races of subjects. Lastly, there was only one athlete who was found to have hypertrophic cardiomyopathy in the study group. Having more number of subjects with true pathology may have aided in increasing validity of the study.

## Conclusion

Despite subtle differences in the Z-scores of the ECG variables in athletes compared to non-athletes, 83% of the athletes had ECG variable Z-scores within the range of -2.5 and 2.5, and 91.1% of the athletes had Z-scores between − 3 and 3. All athletes with normal Z-scores had no sudden cardiac arrest/death on a 6.5-year follow-up. The single athlete with a cardiomyopathy was easily diagnosed with Z-scores exceeding − 3 to 3. ECG assessment in athletes with Z-scores derived from normal subjects may guide clinical decision-making regarding secondary screening, limiting unnecessary referrals to cardiology while providing a sensitive system to capture potential cardiac pathologies. A Z-score based automated ECG screening may be utilized as a first step for pre-participation screening of athletes.

## Electronic Supplementary Material

Below is the link to the electronic supplementary material.

Supplementary Material 1

Supplementary Material 2

Supplementary Material 3

Supplementary Material 4

## Data Availability

The raw data that support the findings of this study are not openly available due to reasons of sensitivity as they involve personal information of participating subjects that may present a risk of reidentification if shared publicly. All secondary data derived from analysis that support the findings of the study are available within the article and its supplemental materials. Deidentified raw data that support the findings of this study may be available from the corresponding author, upon reasonable request.

## Declarations

### Author Information

James C. Perry is an Emeritus Professor for Pediatric Cardiology at University of California San Diego. He was a former Director of Electrophysiology and Adult Congenital Heart Programs at University of California San Diego. He is now a senior consultant in Pediatric Electrophysiology at Stanford University. Andras Bratincsak is an Associate Professor of Pediatrics at John A. Burns School of Medicine at University of Hawaii.

## Abbreviations

ECG: Electrocardiogram
PPE: Pre-participation examination
AHA: American Heart Association
LVH: Left ventricular hypertrophy
HCM: Hypertrophic cardiomyopathy
