# Supplementary material for: Fine Tuning ECG Interpretation for Young Athletes: ECG Screening Using Z-score-based Analysis
Source: Sports Med Open. 2024 Oct 23;10:114. doi: 10.1186/s40798-024-00775-9 (PMC11499507; doi:10.1186/s40798-024-00775-9)
Supplement: Supplementary file 1 — Supplementary Material 1 [file 40798_2024_775_MOESM1_ESM.pdf]

Sports Medicine - Open

Fine tuning ECG interpretation for young athletes: ECG screening using Z-score-based analysis

Authors

Jihyun Park[1,4], Chieko Kimata[2], Justin Young[3], James C Perry[1, 5], Andras Bratincsak[3, 4]

Affiliations

1. University of California San Diego School of Medicine, Department of Pediatrics, San Diego, CA, USA
2. Hawaii Pacific Health, Patient Safety & Quality Services, Honolulu, HI, USA
3. Hawaii Pacific Health Medical Group, Hawaii Pacific Health, Honolulu, HI, USA
4. John A Burns School of Medicine, University of Hawaii, Department of Pediatrics, University of Hawaii, Honolulu, HI, USA
5. Division of Pediatric Cardiology, Stanford University, Stanford, California, USA

Correspondence:

Jihyun Park, MD

3020 Children’s Way

MC5004

San Diego, CA 92123

E-mail: jip019@health.ucsd.edu

Supplementary table 1: Relevant positive answers in the health screening questionnaire for collegiate athletes

Questions/Items			Number of people who answered Yes
Past Medical History	Asthma		6
	Diabetes		0
	Hypertension		1
	Seizures		0
	Other		4
Current Medications (Any cardiac medications including antiarrhythmics, antihypertensives, and/or diuretics? Albuterol PRN is excluded)			1 (Lisinopril)
Cardiac related symptoms/history	Do you get chest pain when you exercise?		7
	Have you ever passed out during or immediately after exercise?		6
	Do you have difficulty breathing or unexplained fatigue during exercise that is new or getting worse?		4
	Does your heart ever race (suddenly beat fast) without good reason?		2
	Have you ever been diagnosed with any of the following conditions?	High blood pressure	1
		Heart infection	0
		High cholesterol	0
		Kawasaki disease	1
		Another heart problem	2 (murmur)
	Has a doctor ever ordered a test for your heart? (for example, ECG/EKG, and/ or Echocardiogram)		5
Family history	Has any of your family died from a heart problem before the age of 50?		4
	Has anyone in your family died suddenly for an unknown reason before the age of 50 (including sudden infant death syndrome (SIDS), unexpected car accident, or drowning)?		1
	Does anyone in your family have any of the following medical problems?	Hypertrophic cardiomyopathy	1
		Dilated cardiomyopathy	0
		Catecholaminergic polymorphic ventricular tachycardia	1
		Brugada syndrome	0
		Arrhythmogenic right ventricular cardiomyopathy (ARVC)	0
		Marfan syndrome	0
		Long QT syndrome	0
		Short QT syndrome	0
		SVT	1
		Unknown heart condition	1
