# Supplementary material for: Fine Tuning ECG Interpretation for Young Athletes: ECG Screening Using Z-score-based Analysis
Source: Sports Med Open. 2024 Oct 23;10:114. doi: 10.1186/s40798-024-00775-9 (PMC11499507; doi:10.1186/s40798-024-00775-9)
Supplement: Supplementary file 2 — Supplementary Material 2 [file 40798_2024_775_MOESM2_ESM.pdf]

Sports Medicine - Open

Fine tuning ECG interpretation for young athletes: ECG screening using Z-score-based analysis

Authors

Jihyun Park[1,4], Chieko Kimata[2], Justin Young[3], James C Perry[1, 5], Andras Bratincsak[3, 4]

Affiliations

1. University of California San Diego School of Medicine, Department of Pediatrics, San Diego, CA, USA
2. Hawaii Pacific Health, Patient Safety & Quality Services, Honolulu, HI, USA
3. Hawaii Pacific Health Medical Group, Hawaii Pacific Health, Honolulu, HI, USA
4. John A Burns School of Medicine, University of Hawaii, Department of Pediatrics, University of Hawaii, Honolulu, HI, USA
5. Division of Pediatric Cardiology, Stanford University, Stanford, California, USA

Correspondence:

Jihyun Park, MD

3020 Children's Way

MC5004

San Diego, CA 92123

E-mail: jip019@health.ucsd.edu

Supplementary table 2: Types of sports and the number of years/hours athletes have played

	Types of Sports	Number of Athletes	Years Played			Hours/week		
			Mean	Median	Mode	Mean	Median	Mode
Female	Golf	7	10.32	11	10	13.31	12	20
	Track	22						
	Volleyball	28						
	Softball	45						
	Water Polo	6						
	Cheer	6						
	Swimming	11						
	Tennis	3						
	Sailing	6						
	Beach Volleyball	5						
	Soccer	56						
	Basketball	15						
	Cross Country	8						
	N/A	106						
	Total	324						
Male	Golf	6	9.68	10	10	14.34	14.5	20
	Volleyball	8						
	Cheer	3						
	Swimming	16						
	Tennis	2						
	Sailing	2						
	Basketball	32						
	Football	163						
	Baseball	26						
	Diving	2						
	N/A	88						
	Total	348						
