# Supplementary material for: Fine Tuning ECG Interpretation for Young Athletes: ECG Screening Using Z-score-based Analysis
Source: Sports Med Open. 2024 Oct 23;10:114. doi: 10.1186/s40798-024-00775-9 (PMC11499507; doi:10.1186/s40798-024-00775-9)
Supplement: Supplementary file 3 — Supplementary Material 3 [file 40798_2024_775_MOESM3_ESM.pdf]

Sports Medicine - Open

Fine tuning ECG interpretation for young athletes: ECG screening using Z-score-based analysis

Authors

Jihyun Park[1,4], Chieko Kimata[2], Justin Young[3], James C Perry[1, 5], Andras Bratincsak[3, 4]

Affiliations

1. University of California San Diego School of Medicine, Department of Pediatrics, San Diego, CA, USA
2. Hawaii Pacific Health, Patient Safety & Quality Services, Honolulu, HI, USA
3. Hawaii Pacific Health Medical Group, Hawaii Pacific Health, Honolulu, HI, USA
4. John A Burns School of Medicine, University of Hawaii, Department of Pediatrics, University of Hawaii, Honolulu, HI, USA
5. Division of Pediatric Cardiology, Stanford University, Stanford, California, USA

Correspondence:

Jihyun Park, MD

3020 Children’s Way

MC5004

San Diego, CA 92123

E-mail: jip019@health.ucsd.edu

Supplementary Figure 1: Distribution of values for ECG variables in controls and athletes by age. UH Athlete refers to the athletes and Non-UH Athlete refers to control cohort. RR interval represents heart rate. A: Distribution of values for RR interval, used as surrogate of heart rate, in athlete and control cohort by age. B: Distribution of values for PR interval in athlete and control cohort by age. C: Distribution of values for R axis, demonstrating degree of axis deviation, in athlete and control cohort by age. D: Distribution of values for QT interval in athlete and control cohort by age. E: Distribution of values for QRS duration in athlete and control cohort by age. F: Distribution of values for T wave peak amplitude in lead II in athlete and control cohort by age. G: Distribution of values for T wave peak amplitude in lead aVF in athlete and control cohort by age. H: Distribution of values for T wave peak amplitude in lead V6 in athlete and control cohort by age. I: Distribution of values for T wave area in lead II in athlete and control cohort by age. J: Distribution of values for T wave area in lead aVF in athlete and control cohort by age. K: Distribution of values for S wave peak amplitude in lead V1 in athlete and control cohort by age. L: Distribution of values for S wave peak amplitude in lead V2 in athlete and control cohort by age. M: Distribution of values for Q wave peak amplitude in lead II in athlete and control cohort by age. N: Distribution of values for Q wave peak amplitude in lead V6 in athlete and control cohort by age. O: Distribution of values for R wave peak amplitude in lead II in athlete and control cohort by age. P: Distribution of values for R wave peak amplitude in lead V6 in athlete and control cohort by age. Q: Distribution of values for R wave peak amplitude in lead V5 in athlete and control cohort by age. R: Distribution of values for R' peak amplitude in lead V1 in athlete and control cohort by age. S: Distribution of values for R' area in lead V1 in athlete and control cohort by age. T: Distribution of values for R' duration in lead V1 in athlete and control cohort by age

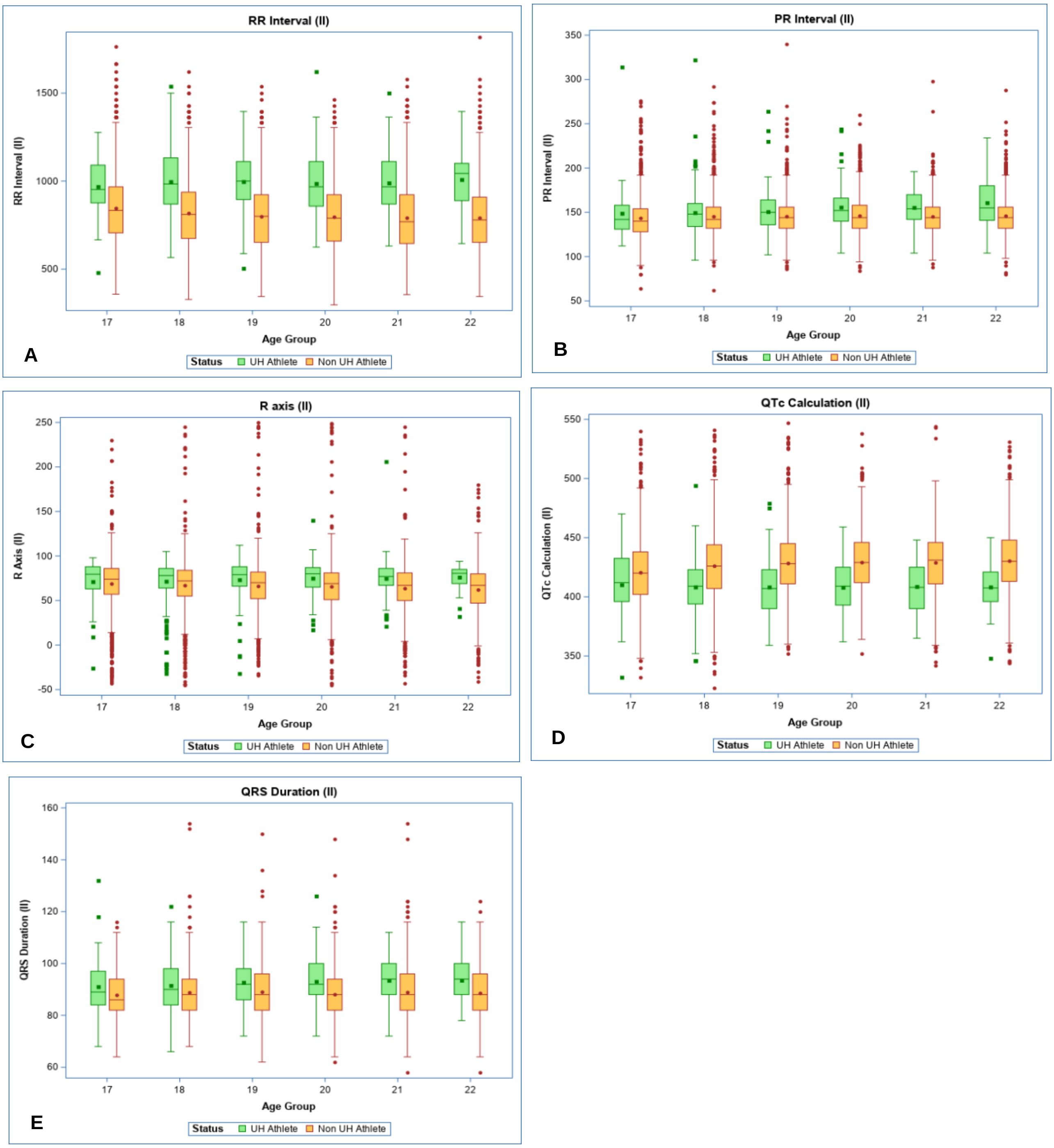
RR Interval (II)
RR Interval (II)
1500
1000
500
17
18
19
20
21
22
Age Group
A
Status
UH Athlete
Non UH Athlete
PR Interval (II)
PR Interval (II)
350
300
250
200
150
100
50
17
18
19
20
21
22
Age Group
B
Status
UH Athlete
Non UH Athlete
R axis (II)
R Axis (II)
250
200
150
100
50
0
-50
17
18
19
20
21
22
Age Group
C
Status
UH Athlete
Non UH Athlete
QTc Calculation (II)
QTc Calculation (II)
550
500
450
400
350
17
18
19
20
21
22
Age Group
D
Status
UH Athlete
Non UH Athlete
QRS Duration (II)
QRS Duration (II)
160
140
120
100
80
60
17
18
19
20
21
22
Age Group
E
Status
UH Athlete
Non UH Athlete

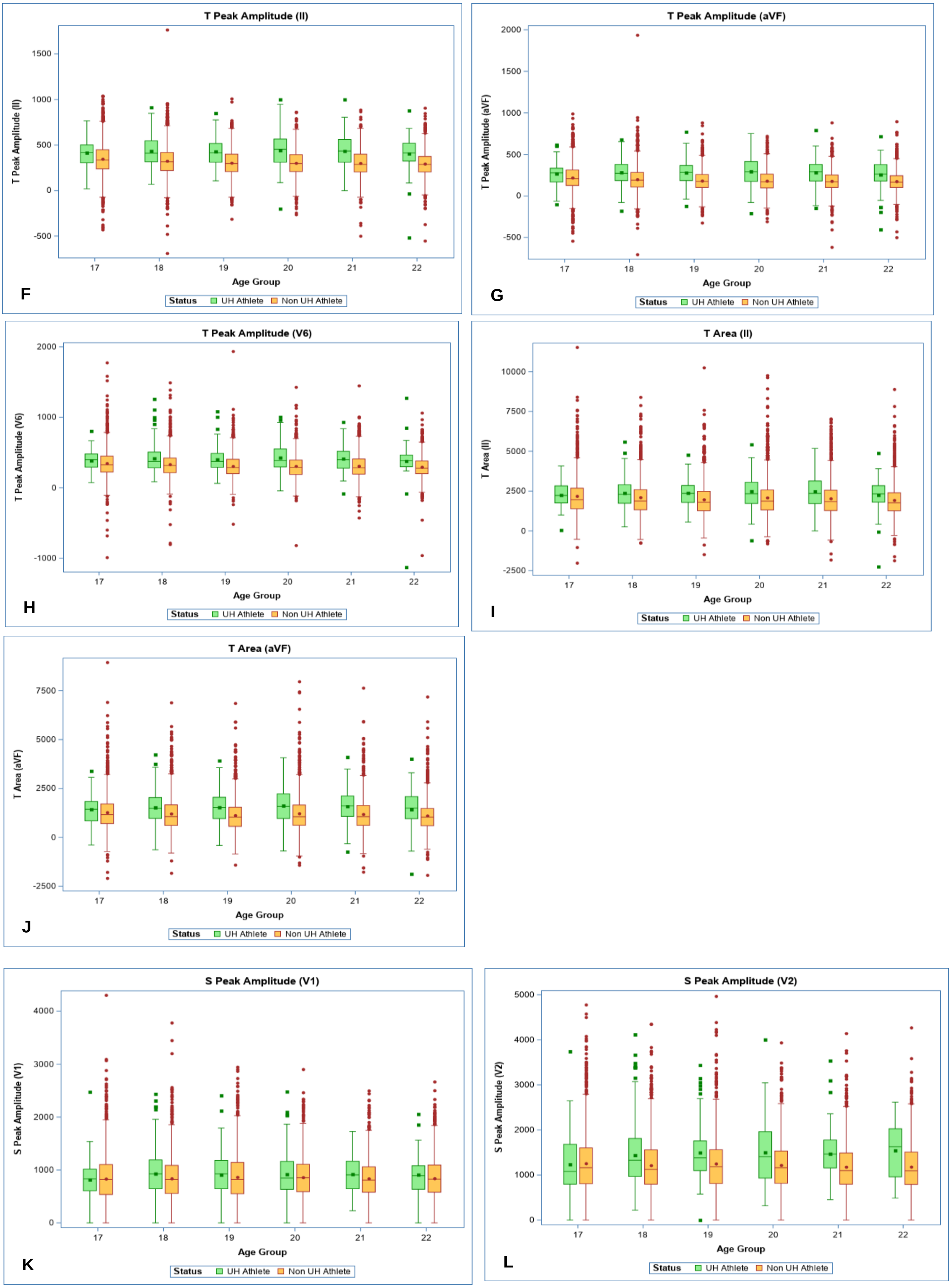

T Peak Amplitude (II)
T Peak Amplitude (II)
Age Group
F
Status UH Athlete Non UH Athlete
T Peak Amplitude (aVF)
T Peak Amplitude (aVF)
Age Group
G
Status UH Athlete Non UH Athlete
T Peak Amplitude (V6)
T Peak Amplitude (V6)
Age Group
H
Status UH Athlete Non UH Athlete
T Area (II)
T Area (II)
Age Group
I
Status UH Athlete Non UH Athlete
T Area (aVF)
T Area (aVF)
Age Group
J
Status UH Athlete Non UH Athlete
S Peak Amplitude (V1)
S Peak Amplitude (V1)
Age Group
K
Status UH Athlete Non UH Athlete
S Peak Amplitude (V2)
S Peak Amplitude (V2)
Age Group
L
Status UH Athlete Non UH Athlete

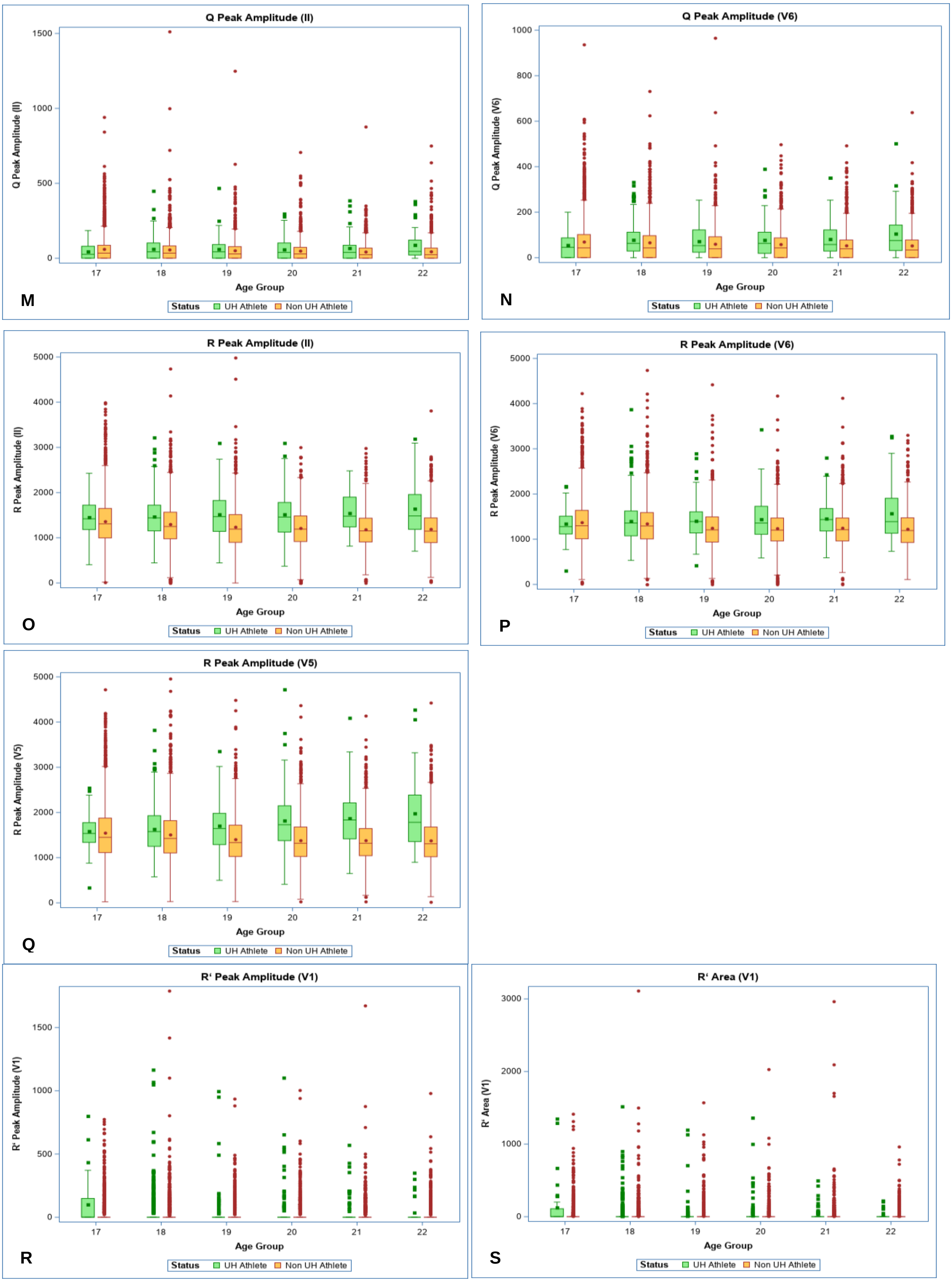

Q Peak Amplitude (II)
Q Peak Amplitude (II)
Age Group
M
Q Peak Amplitude (V6)
Q Peak Amplitude (V6)
Age Group
N
R Peak Amplitude (II)
R Peak Amplitude (II)
Age Group
O
R Peak Amplitude (V6)
R Peak Amplitude (V6)
Age Group
P
R Peak Amplitude (V5)
R Peak Amplitude (V5)
Age Group
Q
R' Peak Amplitude (V1)
R' Peak Amplitude (V1)
Age Group
R
R' Area (V1)
R' Area (V1)
Age Group
S
Status UH Athlete Non UH Athlete

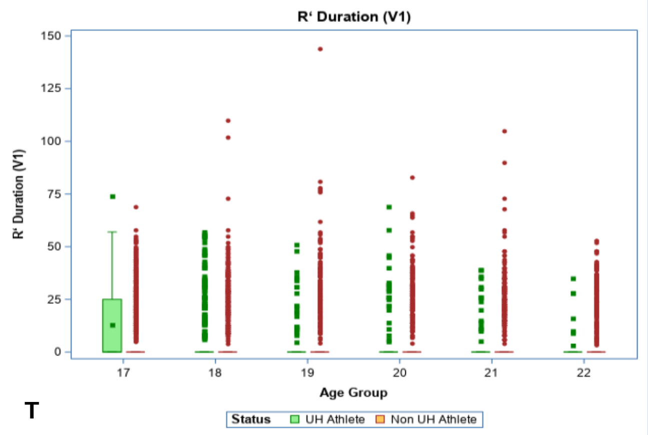
R' Duration (V1)
R' Duration (V1)
Age Group
Status
UH Athlete
Non UH Athlete
150
125
100
75
50
25
0
17
18
19
20
21
22
T
