# Supplementary material for: Fine Tuning ECG Interpretation for Young Athletes: ECG Screening Using Z-score-based Analysis
Source: Sports Med Open. 2024 Oct 23;10:114. doi: 10.1186/s40798-024-00775-9 (PMC11499507; doi:10.1186/s40798-024-00775-9)
Supplement: Supplementary file 4 — Supplementary Material 4 [file 40798_2024_775_MOESM4_ESM.pdf]

Sports Medicine - Open

Fine tuning ECG interpretation for young athletes: ECG screening using Z-score-based analysis

Authors

Jihyun Park[1,4], Chieko Kimata[2], Justin Young[3], James C Perry[1, 5], Andras Bratincsak[3, 4]

Affiliations

1. University of California San Diego School of Medicine, Department of Pediatrics, San Diego, CA, USA
2. Hawaii Pacific Health, Patient Safety & Quality Services, Honolulu, HI, USA
3. Hawaii Pacific Health Medical Group, Hawaii Pacific Health, Honolulu, HI, USA
4. John A Burns School of Medicine, University of Hawaii, Department of Pediatrics, University of Hawaii, Honolulu, HI, USA
5. Division of Pediatric Cardiology, Stanford University, Stanford, California, USA

Correspondence:

Jihyun Park, MD

3020 Children's Way

MC5004

San Diego, CA 92123

E-mail: jip019@health.ucsd.edu

Supplementary Figure 2: Distribution of Z-scores of clinically significant ECG variables in controls and athletes by age and sex. Non-athletes refer to control cohort. A: Distribution of Z-scores for heart rate in athletes and controls by age and sex. B: Distribution of Z-score for PR interval in athletes and controls by age and sex. C: Distribution of Z-score for QTc in athletes and controls by age and sex. D: Distribution of Z-score for R axis in athletes and controls by age and sex. E: Distribution of Z-score for R wave peak amplitude in lead II in athletes and controls by age and sex. F: Distribution of Z-score for T wave peak amplitude in lead II in athletes and controls by age and sex. G: Distribution of Z-score for S wave peak amplitude in lead V2 in athletes and controls by age and sex. H: Distribution of Z-score for R wave peak amplitude in lead V5 in athletes and controls by age and sex. I: Distribution of Z-score for R wave peak amplitude in lead V6 in athletes and controls by age and sex.

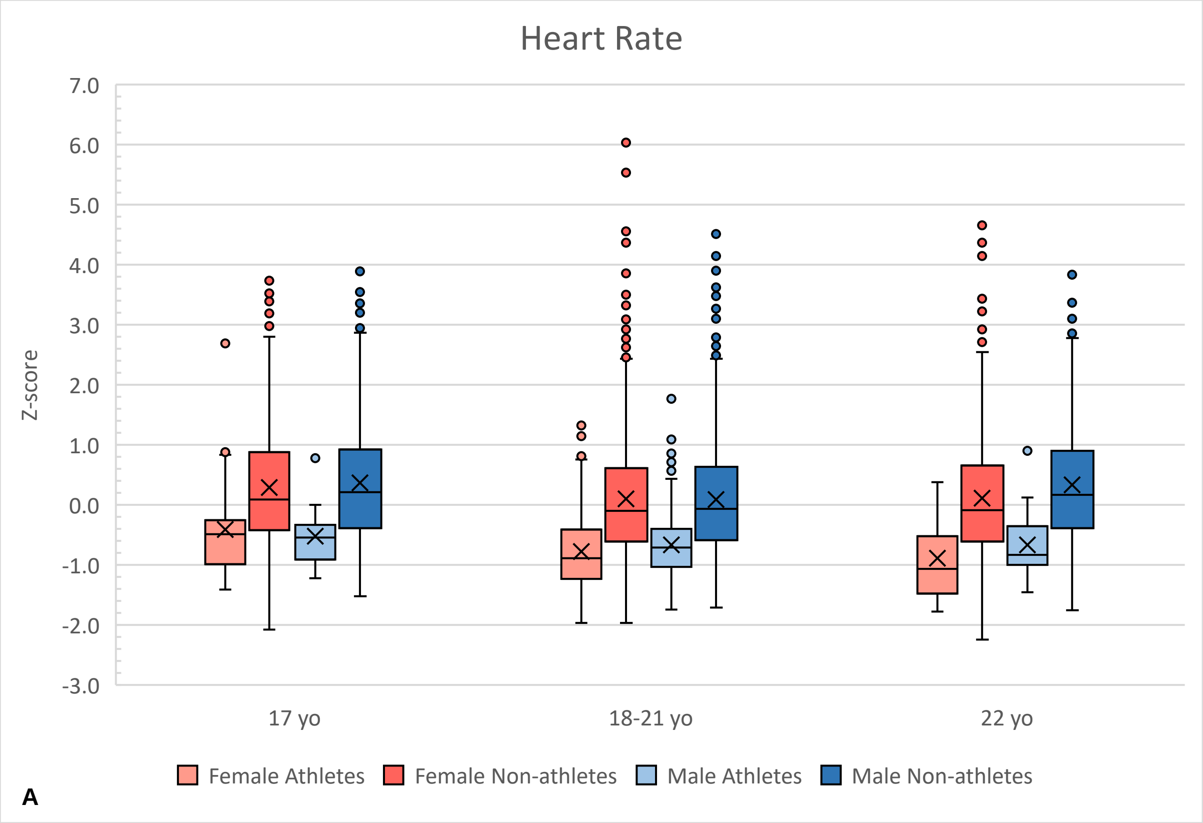
Heart Rate
Z-score
7.0
6.0
5.0
4.0
3.0
2.0
1.0
0.0
-1.0
-2.0
-3.0
17 yo
18-21 yo
22 yo
Female Athletes
Female Non-athletes
Male Athletes
Male Non-athletes
A

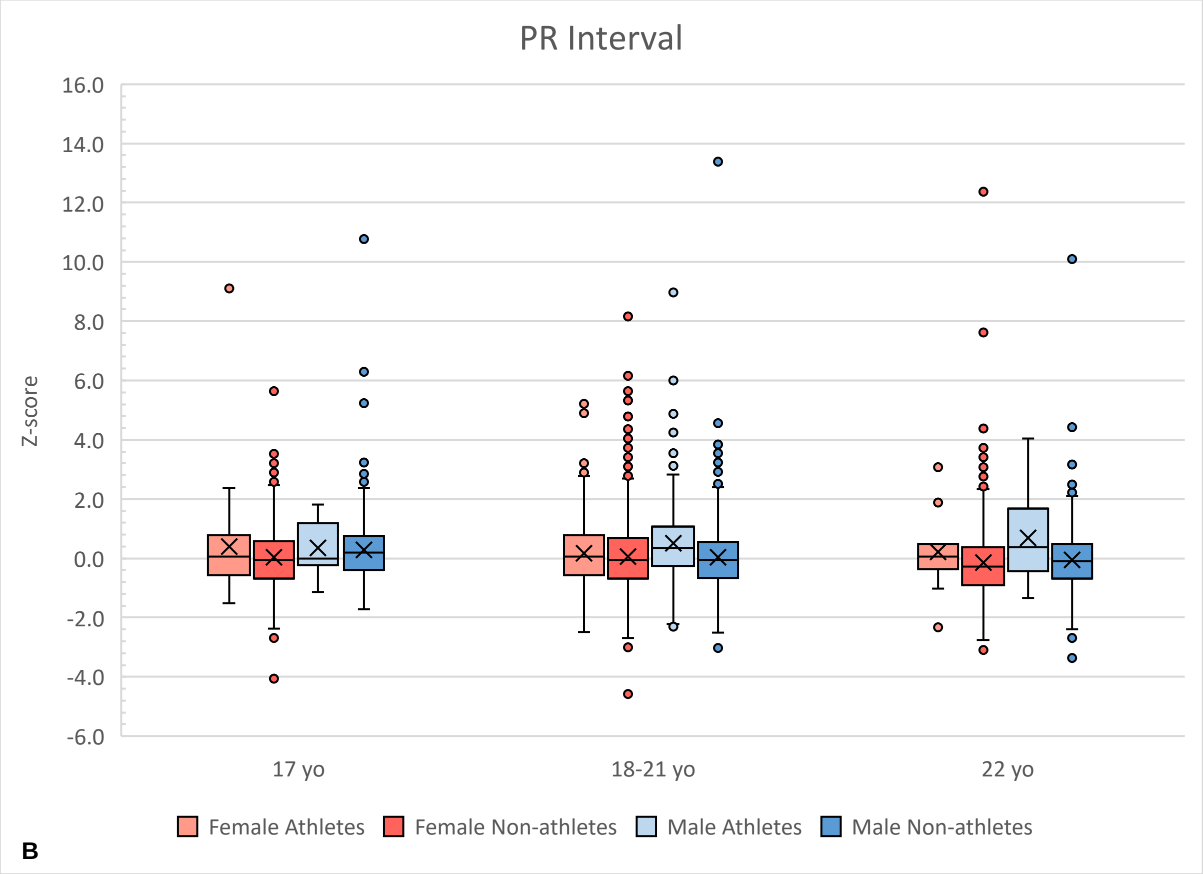
PR Interval
Z-score
16.0
14.0
12.0
10.0
8.0
6.0
4.0
2.0
0.0
-2.0
-4.0
-6.0
17 yo
18-21 yo
22 yo
Female Athletes
Female Non-athletes
Male Athletes
Male Non-athletes
B

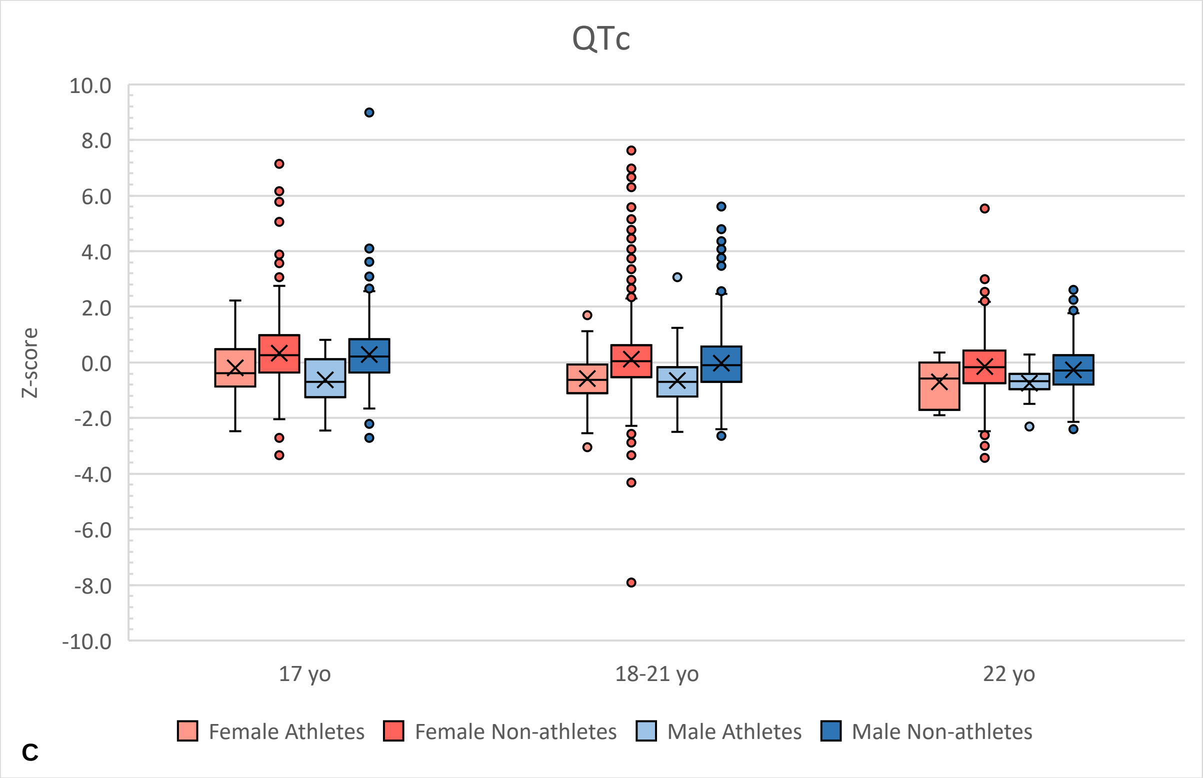
QTc
10.0
8.0
6.0
4.0
2.0
0.0
-2.0
-4.0
-6.0
-8.0
-10.0
Z-score
17 yo
18-21 yo
22 yo
Female Athletes
Female Non-athletes
Male Athletes
Male Non-athletes
C

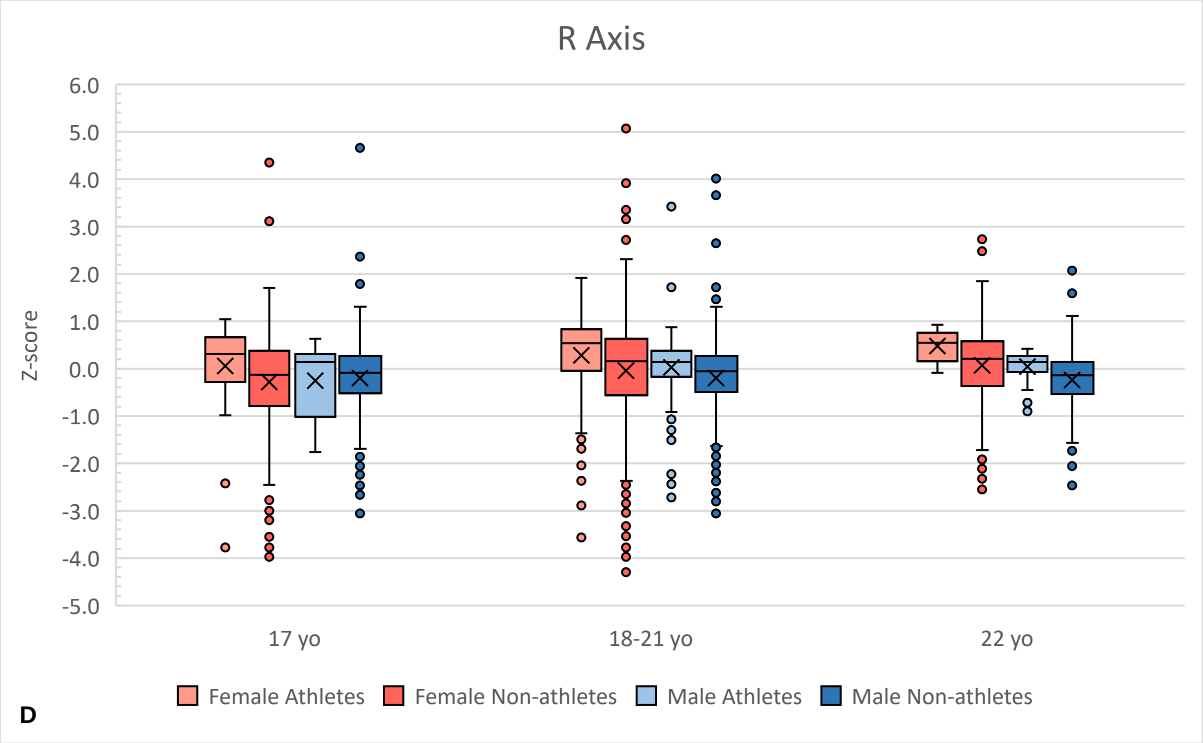
R Axis
6.0
5.0
4.0
3.0
2.0
1.0
0.0
-1.0
-2.0
-3.0
-4.0
-5.0
Z-score
17 yo
18-21 yo
22 yo
Female Athletes
Female Non-athletes
Male Athletes
Male Non-athletes
D

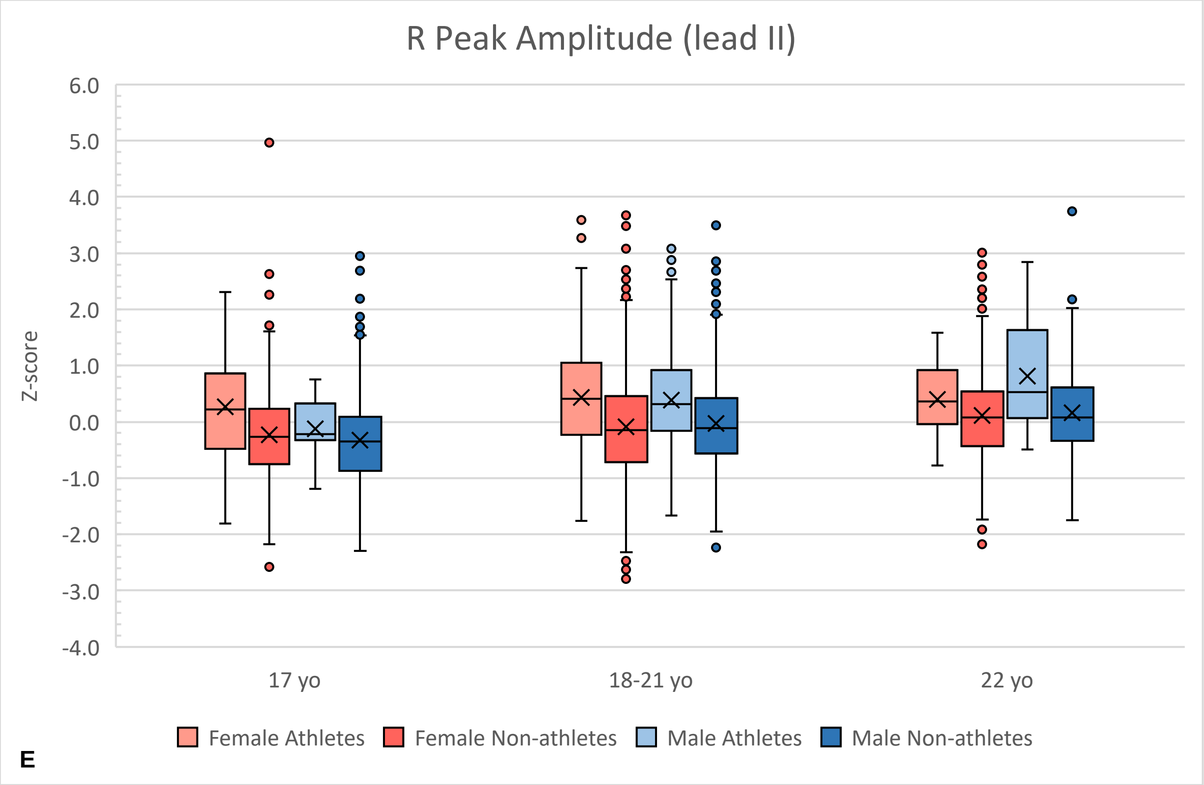
R Peak Amplitude (lead II)
6.0
5.0
4.0
3.0
2.0
1.0
0.0
-1.0
-2.0
-3.0
-4.0
Z-score
17 yo
18-21 yo
22 yo
Female Athletes
Female Non-athletes
Male Athletes
Male Non-athletes
E

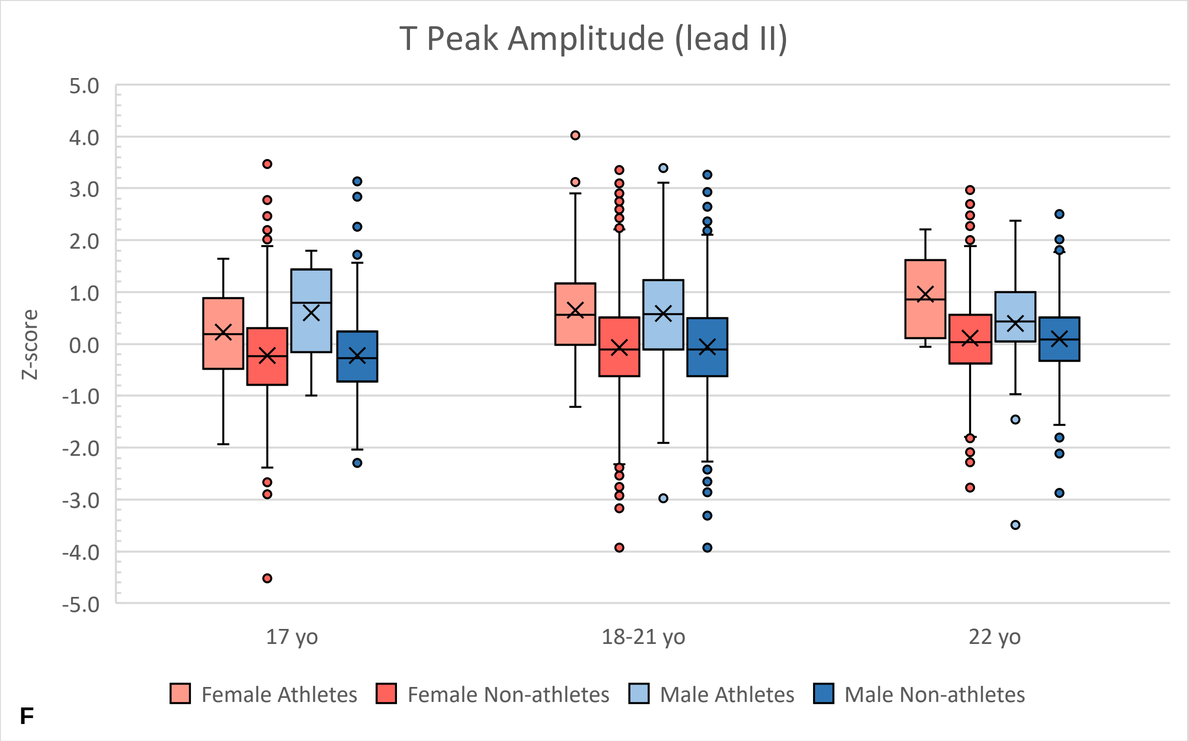
T Peak Amplitude (lead II)
5.0
4.0
3.0
2.0
1.0
0.0
-1.0
-2.0
-3.0
-4.0
-5.0
Z-score
17 yo
18-21 yo
22 yo
Female Athletes
Female Non-athletes
Male Athletes
Male Non-athletes
F

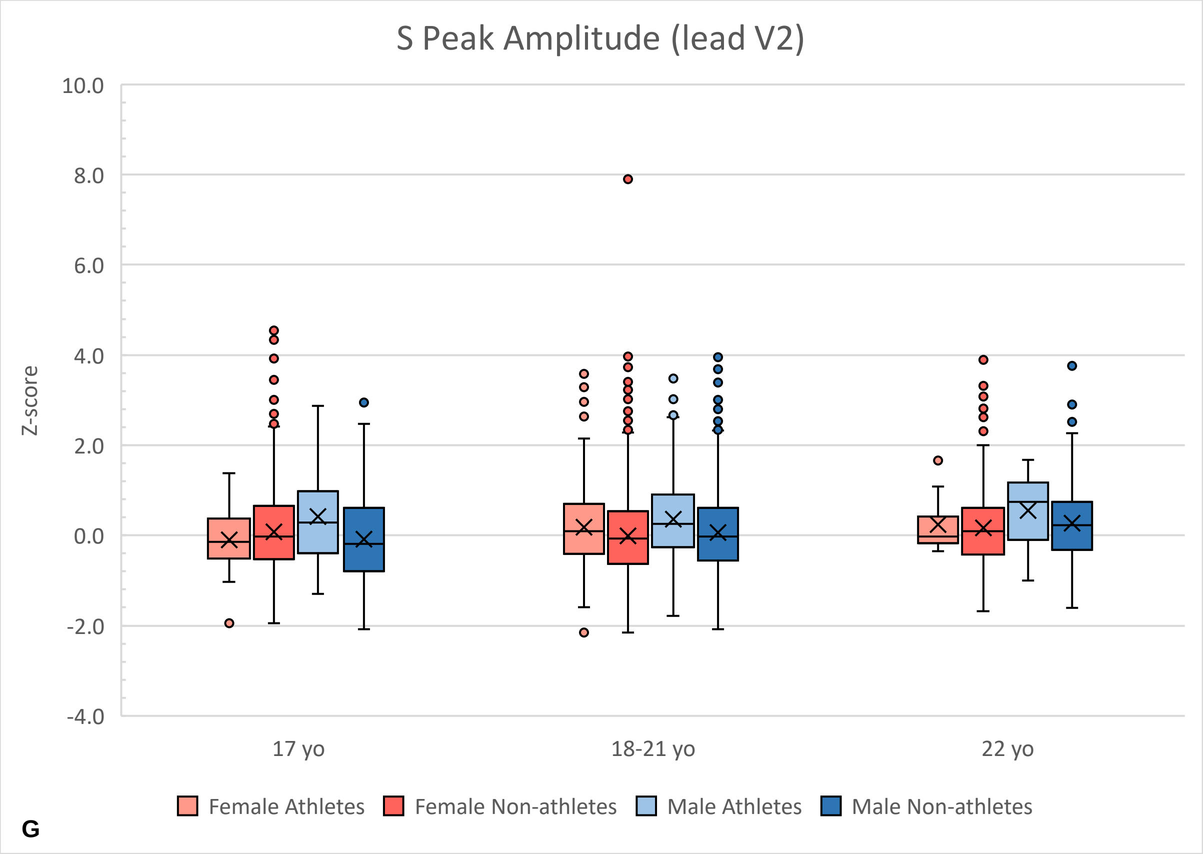
S Peak Amplitude (lead V2)
10.0
8.0
6.0
4.0
2.0
0.0
-2.0
-4.0
Z-score
17 yo
18-21 yo
22 yo
Female Athletes
Female Non-athletes
Male Athletes
Male Non-athletes
G

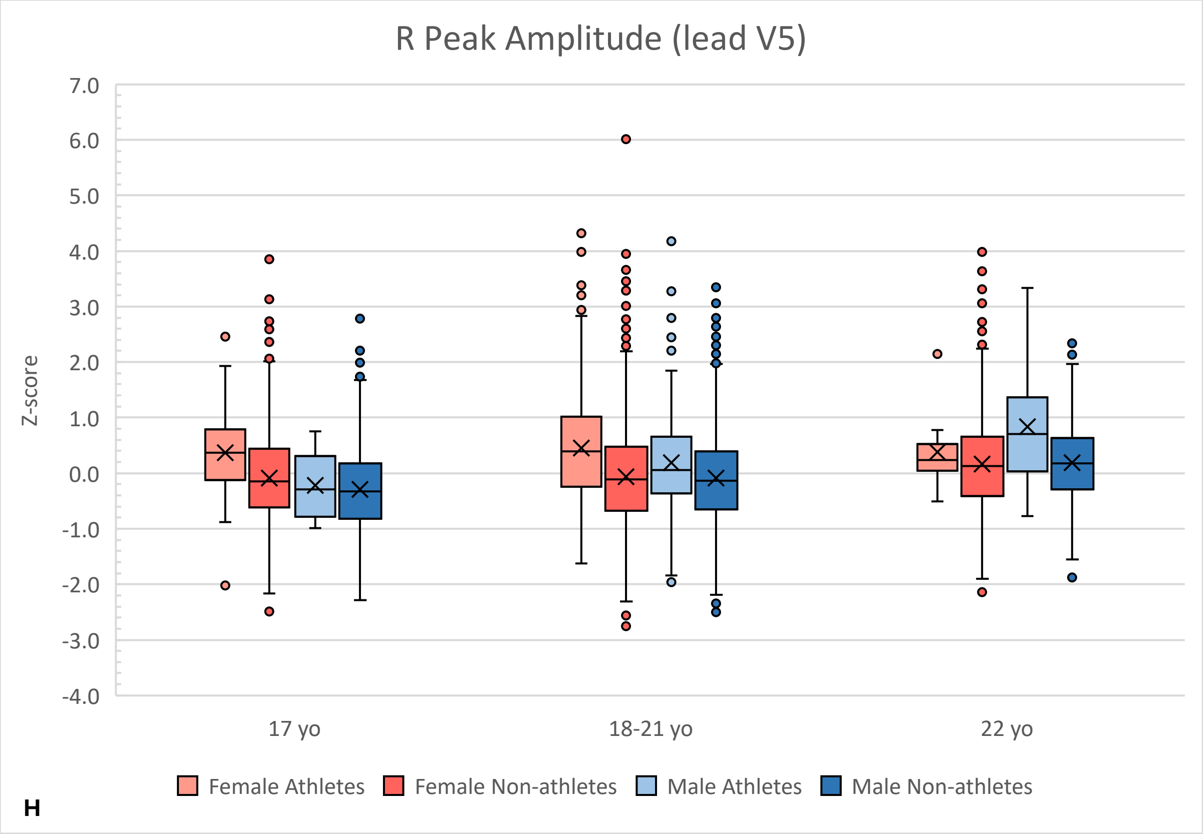
R Peak Amplitude (lead V5)
7.0
6.0
5.0
4.0
3.0
2.0
1.0
0.0
-1.0
-2.0
-3.0
-4.0
Z-score
17 yo
18-21 yo
22 yo
Female Athletes
Female Non-athletes
Male Athletes
Male Non-athletes
H

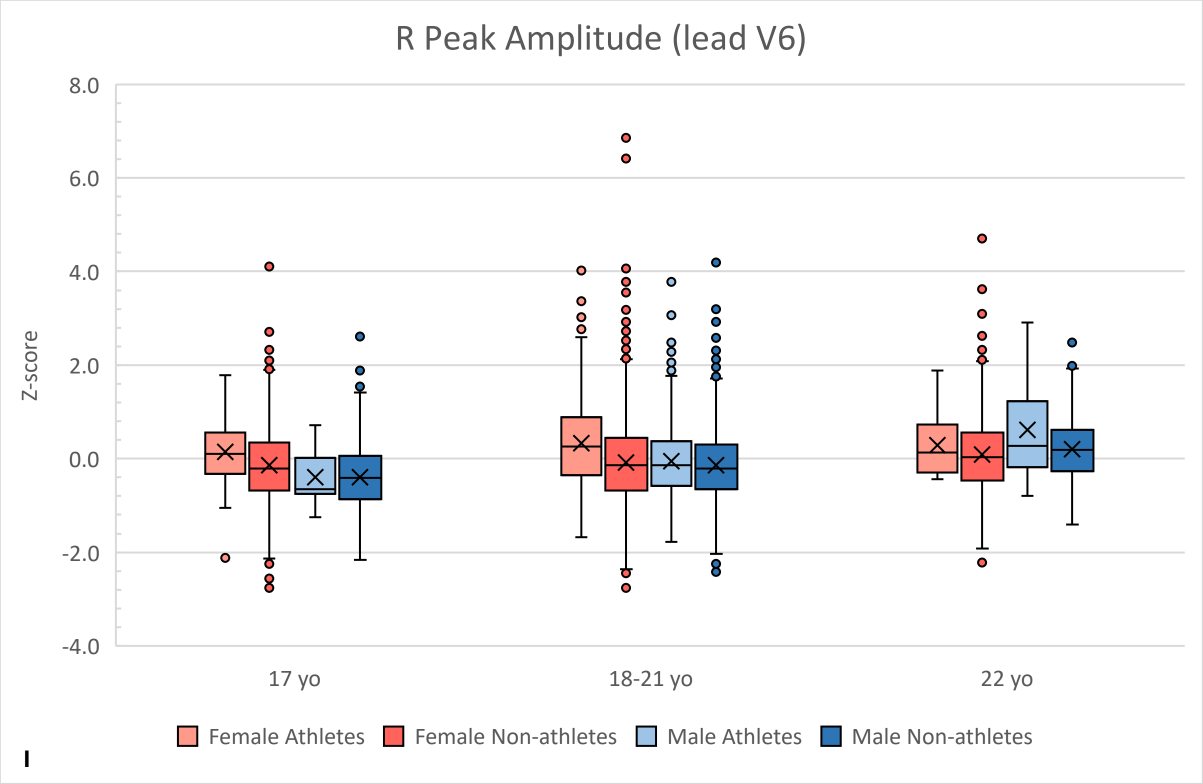
R Peak Amplitude (lead V6)
Z-score
8.0
6.0
4.0
2.0
0.0
-2.0
-4.0
17 yo
18-21 yo
22 yo
Female Athletes
Female Non-athletes
Male Athletes
Male Non-athletes
